# Medical Chemistry

**Published:** 1880-01

**Authors:** 


Medical Chemistry, including the outlines of organic and Physiological Chemistry, based in part upon Riche's Manual of Clinic. By C. Gilbert Wheeler, Prof. of Chemistry in the University of Chicago, and formerly Professor of Organic Chemistry in the Chicago Med College. Second and revised edition. Wm. Wood & Co., New York. 1880.

The demand for this work, it would seem, has caused the necessity for this second edition within one year from the publication of the first, the two being dated 1879 and 1880, respectively.

